# Supplementary material for: Understanding phosphorus dynamics and uptake in diverse soils of Indo-Gangetic Plains of India
Source: PLoS One. 2025 Dec 30;20(12):e0339034. doi: 10.1371/journal.pone.0339034 (PMC12752939; doi:10.1371/journal.pone.0339034)
Supplement: S1 File — (DOCX) [file pone.0339034.s001.docx]

Table S1: Location of soil sampling sites in the study area.

| Sample no. | Location | latitude | longitude |
| --- | --- | --- | --- |
| 1. | Ludhiana-1 | 30.5359 | 75.4741 |
| 2. | Ludhiana-2 | 31.000954 | 75.770886 |
| 3. | Patiala | 30.268217 | 76.389673 |
| 4. | Karnal | 29.711335 | 76.965604 |
| 5. | Hisar | 29.156002 | 75.67911 |
| 6. | Rewari | 28.302433 | 76.697033 |
| 7. | Gurugram | 28.396131 | 76.777702 |
| 8. | Delhi | 28.640848 | 77.154744 |
| 9. | Modipuram | 29.078057 | 77.705439 |
| 10. | Lucknow-1 | 26.5336 | 80.4657 |
| 11. | Lucknow-2 | 26.804357 | 80.931392 |
| 12. | Kanpur | 26.494213 | 80.269003 |
| 13. | Varanasi | 25.303994 | 82.949708 |
| 14. | Mau | 25.897735 | 83.487178 |
| 15. | Buxar | 25.5472 | 83.991111 |
| 16. | Patna | 25.3531 | 85.0507 |
| 17. | Nawada | 24.849237 | 85.888289 |
| 18. | Samastipur | 25.983333 | 85.683333 |
| 19. | Purnea | 25.736767 | 87.509639 |
| 20. | Coochbehar | 26.402771 | 89.391102 |
| 21. | Kalyani | 22.986788 | 88.454095 |
| 22. | Viswabharti | 23.669166 | 87.657941 |
| 23. | Kolkata | 23.232996 | 87.096314 |
| 24. | Barrackpore | 22.759957 | 88.40163 |
| 25. | Uttar Dinajpur | 25.883312 | 87.833562 |

Table S2: The raw values of dataset given in table 2

| R1 | R2 | R3 | Mean |
| --- | --- | --- | --- |
| 4.13 | 4.28 | 4.20 | 4.20 |
| 3.90 | 3.98 | 3.97 | 3.95 |
| 4.22 | 4.38 | 4.20 | 4.27 |
| 4.40 | 4.46 | 4.38 | 4.42 |
| 4.03 | 4.18 | 4.10 | 4.10 |
| 4.42 | 4.54 | 4.50 | 4.49 |
| 4.03 | 4.08 | 4.03 | 4.05 |
| 4.20 | 4.30 | 4.23 | 4.24 |
| 4.44 | 4.55 | 4.41 | 4.47 |
| 4.48 | 4.50 | 4.43 | 4.47 |
| 4.20 | 4.24 | 4.23 | 4.23 |
| 4.42 | 4.56 | 4.51 | 4.50 |
| 4.46 | 4.60 | 4.58 | 4.55 |
| 4.06 | 4.16 | 4.19 | 4.13 |
| 4.50 | 4.31 | 4.38 | 4.40 |
| 4.46 | 4.59 | 4.54 | 4.53 |
| 3.84 | 3.93 | 3.91 | 3.89 |
| 4.38 | 4.35 | 4.39 | 4.37 |
| 3.53 | 3.58 | 3.55 | 3.55 |
| 3.49 | 4.43 | 4.46 | 4.13 |
| 4.41 | 4.39 | 4.38 | 4.39 |
| 4.44 | 4.28 | 4.34 | 4.35 |
| 4.35 | 4.42 | 4.41 | 4.39 |
| 4.55 | 4.45 | 4.51 | 4.50 |
| 4.36 | 4.37 | 4.38 | 4.37 |

Table S3: The raw values of dataset given in table 3

|  | dry wt g/pot | | | | | | | | | |
| --- | --- | --- | --- | --- | --- | --- | --- | --- | --- | --- |
|  |  | Control |  |  |  |  |  | 100% P |  |  |
|  | R1 | R2 | R3 | Mean |  |  | R1 | R2 | R3 | Mean |
| 1 | 11.8 | 10 | 10.9 | 10.9 |  |  | 11.3 | 13.1 | 10.8 | 11.7 |
| 2 | 12.6 | 12.9 | 13.9 | 13.1 |  |  | 14.8 | 12.7 | 12.9 | 13.5 |
| 3 | 13.8 | 9.7 | 12.5 | 12.0 |  |  | 15.3 | 13.2 | 14.8 | 14.4 |
| 4 | 13.6 | 9.9 | 11.4 | 11.6 |  |  | 16 | 12.5 | 12.8 | 13.8 |
| 5 | 12.2 | 10.6 | 10.4 | 11.1 |  |  | 12.2 | 14.4 | 13.6 | 13.4 |
| 6 | 8.4 | 8 | 6.4 | 7.6 |  |  | 13.5 | 13.5 | 9.8 | 12.3 |
| 7 | 12.3 | 13.5 | 12.5 | 12.8 |  |  | 13.9 | 13.5 | 16.3 | 14.6 |
| 8 | 14.2 | 13.3 | 14.7 | 14.1 |  |  | 15.1 | 16.6 | 12.4 | 14.7 |
| 9 | 9.6 | 13.7 | 11.1 | 11.5 |  |  | 11.4 | 12.1 | 12.6 | 12.0 |
| 10 | 5.3 | 7.6 | 6.4 | 6.4 |  |  | 14.7 | 12 | 15 | 13.9 |
| 11 | 3.2 | 1.8 | 3.1 | 2.7 |  |  | 14.3 | 17.1 | 12.3 | 14.6 |
| 12 | 12 | 12.6 | 15.2 | 13.3 |  |  | 14.9 | 15 | 14.4 | 14.8 |
| 13 | 13.5 | 14.2 | 14.9 | 14.2 |  |  | 15.4 | 14 | 13.3 | 14.2 |
| 14 | 9.5 | 14.4 | 11 | 11.6 |  |  | 15.3 | 16 | 17.8 | 16.4 |
| 15 | 13.3 | 13 | 13.6 | 13.3 |  |  | 14.8 | 14.7 | 14.7 | 14.7 |
| 16 | 15.2 | 14.3 | 15.2 | 14.9 |  |  | 15.6 | 15.4 | 16.4 | 15.8 |
| 17 | 15.1 | 14.1 | 13.7 | 14.3 |  |  | 14.6 | 14.5 | 15.1 | 14.7 |
| 18 | 4.4 | 6 | 5.5 | 5.3 |  |  | 15.2 | 15.1 | 14.6 | 15.0 |
| 19 | 14.2 | 14.5 | 11.3 | 13.3 |  |  | 15.4 | 14.8 | 18 | 16.1 |
| 20 | 14.6 | 13.6 | 18.5 | 15.6 |  |  | 14.6 | 16.3 | 17.4 | 16.1 |
| 21 | 13 | 10.6 | 10.9 | 11.5 |  |  | 15.8 | 12.6 | 13 | 13.8 |
| 22 | 10.7 | 11 | 11.3 | 11.0 |  |  | 11.9 | 10.8 | 11.7 | 11.5 |
| 23 | 9.3 | 12.1 | 10.8 | 10.7 |  |  | 14.9 | 12.1 | 13.6 | 13.5 |
| 24 | 12 | 14.2 | 12.6 | 12.9 |  |  | 15 | 14.5 | 18.2 | 15.9 |
| 25 | 12.8 | 11.6 | 12 | 12.1 |  |  | 16.4 | 17 | 13.9 | 15.8 |
| Mean | 11.464 | 11.488 | 11.592 | 11.5 |  |  | 14.492 | 14.14 | 14.216 | 14.3 |

|  |  | P conc. mg/g/pot | | | | | | | | |
| --- | --- | --- | --- | --- | --- | --- | --- | --- | --- | --- |
|  |  | Control |  |  |  |  |  | 100% P |  |  |
|  | R1 | R2 | R3 | Mean |  |  | R1 | R2 | R3 | Mean |
| 1 | 2.32 | 1.85 | 2.13 | 2.10 |  |  | 2.26 | 2.60 | 2.19 | 2.35 |
| 2 | 2.20 | 1.95 | 1.90 | 2.02 |  |  | 2.02 | 2.75 | 1.65 | 2.14 |
| 3 | 1.14 | 0.83 | 1.24 | 1.07 |  |  | 0.80 | 1.25 | 1.45 | 1.17 |
| 4 | 0.90 | 0.94 | 1.12 | 0.99 |  |  | 1.37 | 1.55 | 1.54 | 1.49 |
| 5 | 1.74 | 1.72 | 1.44 | 1.63 |  |  | 2.58 | 2.46 | 2.57 | 2.54 |
| 6 | 1.71 | 1.30 | 1.44 | 1.48 |  |  | 1.57 | 1.39 | 2.12 | 1.70 |
| 7 | 1.39 | 1.53 | 2.00 | 1.64 |  |  | 1.65 | 2.56 | 1.97 | 2.06 |
| 8 | 2.11 | 1.58 | 1.73 | 1.81 |  |  | 1.77 | 2.57 | 2.11 | 2.15 |
| 9 | 0.74 | 0.51 | 0.66 | 0.64 |  |  | 2.59 | 2.45 | 3.14 | 2.73 |
| 10 | 0.74 | 0.59 | 0.57 | 0.63 |  |  | 1.13 | 0.87 | 0.94 | 0.98 |
| 11 | 0.47 | 0.29 | 0.35 | 0.37 |  |  | 0.38 | 0.49 | 0.56 | 0.48 |
| 12 | 1.56 | 1.14 | 1.12 | 1.27 |  |  | 2.77 | 2.47 | 2.34 | 2.53 |
| 13 | 2.20 | 2.45 | 2.42 | 2.36 |  |  | 3.02 | 2.99 | 2.33 | 2.78 |
| 14 | 1.25 | 1.48 | 1.51 | 1.41 |  |  | 1.32 | 1.44 | 1.64 | 1.46 |
| 15 | 2.59 | 2.44 | 2.77 | 2.60 |  |  | 3.01 | 3.48 | 3.35 | 3.28 |
| 16 | 1.27 | 1.19 | 1.22 | 1.23 |  |  | 1.59 | 1.38 | 1.46 | 1.48 |
| 17 | 1.80 | 1.39 | 1.66 | 1.62 |  |  | 2.71 | 2.30 | 2.22 | 2.41 |
| 18 | 0.95 | 0.94 | 0.89 | 0.93 |  |  | 1.27 | 1.57 | 1.39 | 1.41 |
| 19 | 2.84 | 2.63 | 2.41 | 2.63 |  |  | 3.23 | 2.98 | 3.67 | 3.29 |
| 20 | 1.45 | 1.75 | 1.21 | 1.47 |  |  | 1.62 | 1.73 | 1.36 | 1.57 |
| 21 | 1.08 | 1.06 | 0.93 | 1.02 |  |  | 1.61 | 1.92 | 1.91 | 1.81 |
| 22 | 0.96 | 1.11 | 0.81 | 0.96 |  |  | 1.10 | 1.18 | 0.87 | 1.05 |
| 23 | 3.15 | 3.15 | 2.79 | 3.03 |  |  | 3.39 | 3.73 | 3.59 | 3.57 |
| 24 | 2.00 | 1.92 | 2.21 | 2.05 |  |  | 2.07 | 2.24 | 2.41 | 2.24 |
| 25 | 1.09 | 0.94 | 1.18 | 1.07 |  |  | 1.54 | 1.56 | 1.72 | 1.61 |
|  | 1.59 | 1.47 | 1.51 | 1.52 |  |  | 1.93 | 2.08 | 2.02 | 2.01 |

Table S4 The raw values of dataset given in table 4

| P/Zn ratio | |  |  |
| --- | --- | --- | --- |
| Treat | Rep | Control | 100% P |
| 1 | 1 | 309 | 357 |
| 2 | 1 | 119 | 415 |
| 3 | 1 | 172 | 175 |
| 4 | 1 | 79 | 89 |
| 5 | 1 | 150 | 199 |
| 6 | 1 | 124 | 359 |
| 7 | 1 | 233 | 350 |
| 8 | 1 | 171 | 185 |
| 9 | 1 | 77 | 258 |
| 10 | 1 | 93 | 180 |
| 11 | 1 | 70 | 97 |
| 12 | 1 | 48 | 178 |
| 13 | 1 | 29 | 343 |
| 14 | 1 | 223 | 324 |
| 15 | 1 | 64 | 132 |
| 16 | 1 | 63 | 109 |
| 17 | 1 | 202 | 161 |
| 18 | 1 | 223 | 301 |
| 19 | 1 | 183 | 203 |
| 20 | 1 | 137 | 227 |
| 21 | 1 | 200 | 179 |
| 22 | 1 | 254 | 252 |
| 23 | 1 | 103 | 228 |
| 24 | 1 | 280 | 178 |
| 25 | 1 | 212 | 1743 |
| 1 | 2 | 237 | 339 |
| 2 | 2 | 105 | 396 |
| 3 | 2 | 129 | 169 |
| 4 | 2 | 66 | 89 |
| 5 | 2 | 129 | 170 |
| 6 | 2 | 129 | 331 |
| 7 | 2 | 244 | 327 |
| 8 | 2 | 195 | 309 |
| 9 | 2 | 86 | 261 |
| 10 | 2 | 95 | 186 |
| 11 | 2 | 60 | 98 |
| 12 | 2 | 40 | 185 |
| 13 | 2 | 19 | 363 |
| 14 | 2 | 204 | 362 |
| 15 | 2 | 84 | 159 |
| 16 | 2 | 43 | 104 |
| 17 | 2 | 191 | 167 |
| 18 | 2 | 214 | 307 |
| 19 | 2 | 179 | 201 |
| 20 | 2 | 106 | 261 |
| 21 | 2 | 147 | 184 |
| 22 | 2 | 204 | 269 |
| 23 | 2 | 76 | 253 |
| 24 | 2 | 303 | 190 |
| 25 | 2 | 256 | 1761 |
| 1 | 3 | 255 | 312 |
| 2 | 3 | 100 | 395 |
| 3 | 3 | 155 | 179 |
| 4 | 3 | 63 | 82 |
| 5 | 3 | 158 | 190 |
| 6 | 3 | 116 | 479 |
| 7 | 3 | 234 | 326 |
| 8 | 3 | 143 | 222 |
| 9 | 3 | 95 | 309 |
| 10 | 3 | 76 | 147 |
| 11 | 3 | 81 | 109 |
| 12 | 3 | 41 | 173 |
| 13 | 3 | 24 | 341 |
| 14 | 3 | 183 | 333 |
| 15 | 3 | 84 | 150 |
| 16 | 3 | 62 | 109 |
| 17 | 3 | 184 | 177 |
| 18 | 3 | 205 | 296 |
| 19 | 3 | 164 | 222 |
| 20 | 3 | 166 | 257 |
| 21 | 3 | 181 | 204 |
| 22 | 3 | 220 | 260 |
| 23 | 3 | 73 | 239 |
| 24 | 3 | 297 | 190 |
| 25 | 3 | 188 | 1950 |
