# Supplementary material for: Understanding phosphorus dynamics and uptake in diverse soils of Indo-Gangetic Plains of India
Source: PLoS One. 2025 Dec 30;20(12):e0339034. doi: 10.1371/journal.pone.0339034 (PMC12752939; doi:10.1371/journal.pone.0339034)
Supplement: Attachment — Submitted filename: PONE-D-25-53866_reviewer.pdf [file pone.0339034.s003.pdf]

PLOS ONE

Understanding phosphorus dynamics and uptake in diverse soils of Indo-Gangetic Plains of India

--Manuscript Draft--

Manuscript Number:	PONE-D-25-53866
Article Type:	Research Article
Full Title:	Understanding phosphorus dynamics and uptake in diverse soils of Indo-Gangetic Plains of India
Short Title:	Phopshorus dynamics and uptake in soils of IGP
Corresponding Author:	koteswara Rao Karnena<br>Central Rice Research Institute: ICAR - National Rice Research Institute<br>Srikakulam, INDIA
Keywords:	Phosphorus availability; nutrient uptake; Indo-Gangetic Plains; nutrient interactions; phosphate potential
Abstract:	Phosphorus (P) is a critical macronutrient governing crop productivity, yet its availability in soils is limited by soil physico-chemical properties and interactions with other nutrients. The present study investigated phosphorus dynamics and uptake by wheat grown on twenty-five soils collected from different locations across the Indo-Gangetic Plains (IGP) of India. The soils exhibited wide variation in texture, pH, organic carbon, and available macro and micro nutrients. Phosphate ion concentration and phosphate potential varied significantly (p<0.05), reflecting contrasting P-supplying capacities. A pot experiment was conducted with wheat under control (no P) and 100% recommended P fertilizer application. Significant variation (p<0.05) was observed in dry matter yield, P concentration, and P uptake among soils and treatments. Fertilizer P application enhanced dry matter accumulation by 10–70% depending on the soil, and P uptake increased more than twice in responsive soils. Particularly, excessive P application altered Zn concentration and the P/Zn ratio in wheat straw, indicating nutrient interactions. The results highlight that soil variability across the IGP governs P availability and wheat response, with implications for site-specific nutrient management to enhance productivity and minimize P-use inefficiencies.
Order of Authors:	koteswara Rao Karnena
	Mandira Barman
	Debarup Das
	Vinod Kumar Sharma
	Debrup Ghosh
	H. Krishna
	T.K. Das
Additional Information:	
Question	Response
**Financial Disclosure**<br><br>Enter a financial disclosure statement that describes the sources of funding for the work included in this submission. Review the submission guidelines for detailed requirements. View published research articles from *PLOS ONE* for specific examples.	Yes

Powered by Editorial Manager® and ProduXion Manager® from Aries Systems Corporation

This statement is required for submission and **will appear in the published article** if the submission is accepted. Please make sure it is accurate.<br><br>**Funded studies**<br>Enter a statement with the following details:<br>• Initials of the authors who received each award<br>• Grant numbers awarded to each author<br>• The full name of each funder<br>• URL of each funder website<br>• Did the sponsors or funders play any role in the study design, data collection and analysis, decision to publish, or preparation of the manuscript?<br><br>Did you receive funding for this work?	
Please add funding details.<br>as follow-up to "**Financial Disclosure**<br><br>Enter a financial disclosure statement that describes the sources of funding for the work included in this submission. Review the submission guidelines for detailed requirements. View published research articles from *PLOS ONE* for specific examples.<br><br>This statement is required for submission and **will appear in the published article** if the submission is accepted. Please make sure it is accurate.<br><br>**Funded studies**<br>Enter a statement with the following details:<br>• Initials of the authors who received each award<br>• Grant numbers awarded to each author<br>• The full name of each funder<br>• URL of each funder website<br>• Did the sponsors or funders play any role in the study design, data collection and analysis, decision to publish, or preparation of the manuscript?	Part of the research was supported by a grant from the Bill & Melinda Gates Foundation (Grant 467 number # OPP1215722) sub-grant to India for the Zn mainstreaming project. The funders had no role in study design, data collection and analysis, decision to publish, or preparation of the manuscript.

Powered by Editorial Manager® and ProduXion Manager® from Aries Systems Corporation

Did you receive funding for this work?"	
Please select the country of your main research funder (please select carefully as in some cases this is used in fee calculation).<br>as follow-up to "**Financial Disclosure**<br><br>Enter a financial disclosure statement that describes the sources of funding for the work included in this submission. Review the submission guidelines for detailed requirements. View published research articles from *PLOS ONE* for specific examples.<br><br>This statement is required for submission and **will appear in the published article** if the submission is accepted. Please make sure it is accurate.<br><br>**Funded studies**<br>Enter a statement with the following details:<br>• Initials of the authors who received each award<br>• Grant numbers awarded to each author<br>• The full name of each funder<br>• URL of each funder website<br>• Did the sponsors or funders play any role in the study design, data collection and analysis, decision to publish, or preparation of the manuscript?<br><br>Did you receive funding for this work?"	UNITED STATES - US
**Competing Interests**<br><br>Use the instructions below to enter a competing interest statement for this submission. On behalf of all authors, disclose any competing interests that could be perceived to bias this work—acknowledging all financial support and any other relevant financial or non-financial competing interests.<br><br>This statement is required for submission and **will appear in the published article** if	The authors have declared that no competing interests exist.

the submission is accepted. Please make sure it is accurate and that any funding sources listed in your Funding Information later in the submission form are also declared in your Financial Disclosure statement.<br><br>View published research articles from *PLOS ONE* for specific examples.<br><br>**NO authors have competing interests**<br><br>Enter: *The authors have declared that no competing interests exist.*<br><br>**Authors with competing interests**<br><br>Enter competing interest details beginning with this statement:<br><br>*I have read the journal's policy and the authors of this manuscript have the following competing interests: [insert competing interests here]*<br><br>* typeset	
**Ethics Statement**<br><br>Enter an ethics statement for this submission. This statement is required if the study involved:<br><br>• Human participants<br>• Human specimens or tissue<br>• Vertebrate animals or cephalopods<br>• Vertebrate embryos or tissues<br>• Field research<br><br>Write "N/A" if the submission does not require an ethics statement.<br><br>General guidance is provided below. Consult the submission guidelines for detailed instructions. **Make sure that all information entered here is included in the Methods section of the manuscript.**	This study does not require collection of plant, animal or other material from a natural setting.

Powered by Editorial Manager® and ProduXion Manager® from Aries Systems Corporation

**Format for specific study types**<br><br>**Human Subject Research (involving human participants and/or tissue)**<br>• Give the name of the institutional review board or ethics committee that approved the study<br>• Include the approval number and/or a statement indicating approval of this research<br>• Indicate the form of consent obtained (written/oral) or the reason that consent was not obtained (e.g. the data were analyzed anonymously)<br><br>**Animal Research (involving vertebrate animals, embryos or tissues)**<br>• Provide the name of the Institutional Animal Care and Use Committee (IACUC) or other relevant ethics board that reviewed the study protocol, and indicate whether they approved this research or granted a formal waiver of ethical approval<br>• Include an approval number if one was obtained<br>• If the study involved *non-human primates*, add *additional details* about animal welfare and steps taken to ameliorate suffering<br>• If anesthesia, euthanasia, or any kind of animal sacrifice is part of the study, include briefly which substances and/or methods were applied<br><br>**Field Research**<br><br>Include the following details if this study involves the collection of plant, animal, or other materials from a natural setting:<br>• Field permit number<br>• Name of the institution or relevant body that granted permission	
**Data Availability**<br><br>Authors are required to make all data underlying the findings described fully available, without restriction, and from the time of publication. PLOS allows rare exceptions to address legal and ethical concerns. See the PLOS Data Policy and FAQ for detailed information.	Yes - all data are fully available without restriction

A Data Availability Statement describing where the data can be found is required at submission. Your answers to this question constitute the Data Availability Statement and **will be published in the article**, if accepted.<br><br>**Important:** Stating 'data available on request from the author' is not sufficient. If your data are only available upon request, select 'No' for the first question and explain your exceptional situation in the text box.<br><br>Do the authors confirm that all data underlying the findings described in their manuscript are fully available without restriction?	
**Describe where the data may be found in full sentences. If you are copying our sample text, replace any instances of XXX with the appropriate details.**<br><br>• If the data are **held or will be held in a public repository**, include URLs, accession numbers or DOIs. If this information will only be available after acceptance, indicate this by ticking the box below. For example: *All XXX files are available from the XXX database (accession number(s) XXX, XXX.*).<br>• If the data are all contained **within the manuscript and/or Supporting Information files**, enter the following: *All relevant data are within the manuscript and its Supporting Information files.*<br>• If neither of these applies but you are able to provide **details of access elsewhere**, with or without limitations, please do so. For example:<br><br>*Data cannot be shared publicly because of [XXX]. Data are available from the XXX Institutional Data Access / Ethics Committee (contact via XXX) for researchers who meet the criteria for access to confidential data.*<br><br>*The data underlying the results presented in the study are available from (include the name of the third party*	All relevant data are within the manuscript and supporting information files.

and contact information or URL).<br>• This text is appropriate if the data are owned by a third party and authors do not have permission to share the data.<br><br>* typeset	
Additional data availability information:	

Manuscript Click here to access/download;Manuscript;Manuscript.docx

Understanding phosphorus dynamics and uptake in diverse soils of Indo-Gangetic Plains of India

Short Title: Phopshorus dynamics and uptake in soils of IGP

K. K. Rao[1,2]*, Mandira Barman[2]*, Debarup Das[2], Vinod Kumar Sharma[2], Debrup Ghosh[2], H. Krishna[2] and T.K. Das[2]

[1,2] Regional Coastal Rice Research Station, ICAR Central rice Research Institute, Srikakulam, Andhra Pradesh-532185

[2] ICAR-Indian Agricultural Research Institute, New Delhi-110012

*Corresponding author e-mail: Koti1012@gmail.com

mandira.ssaciari@gmail.com

Understanding phosphorus dynamics and uptake in diverse soils of Indo-Gangetic Plains of India

Abstract

Phosphorus (P) is a critical macronutrient governing crop productivity, yet its availability in soils is limited by soil physico-chemical properties and interactions with other nutrients. The present study investigated phosphorus dynamics and uptake by wheat grown on twenty-five soils collected from different locations across the Indo-Gangetic Plains (IGP) of India. The soils exhibited wide variation in texture, pH, organic carbon, and available macro and micro nutrients. Phosphate ion concentration and phosphate potential varied significantly ($p<0.05$), reflecting contrasting P-supplying capacities. A pot experiment was conducted with wheat under control (no P) and 100% recommended P fertilizer application. Significant variation ($p<0.05$) was observed in dry matter yield, P concentration, and P uptake among soils and treatments. Fertilizer P application enhanced dry matter accumulation by 10–70% depending on the soil, and P uptake increased more than twice in responsive soils. Particularly, excessive P application altered Zn concentration and the P/Zn ratio in wheat straw, indicating nutrient interactions. The results highlight that soil variability across the IGP governs P availability and wheat response, with implications for site-specific nutrient management to enhance productivity and minimize P-use inefficiencies.

Keywords: Phosphorus availability, nutrient uptake, Indo-Gangetic Plains, nutrient interactions, phosphate potential

Introduction

Wheat (*Triticum aestivum* L.) is the second most crucial staple food crop in India, following rice, covering around 30 million hectares with yearly production surpassing 110 million tonnes [1,2]. The Indo-Gangetic Plains (IGP), extending from Punjab and Haryana in the west across Uttar Pradesh and Bihar to West Bengal in the east, forms the primary wheat-producing region of the nation, contributes over 70% of the country's total wheat production [3]. This region supports the food security of over half a billion people, and its productivity is critical not only for India but also for global grain supplies. Maintaining wheat output in the IGP necessitates a thoughtful balance between resource utilization efficiency, soil health, and nutrient administration. Among the essential nutrients, Phosphorus (P) is one of the vital nutrients that significantly impacts early crop establishment, root growth, tillering, and grain development [4].

The accessibility of this nutrient to plants is regulated by intricate soil chemical interactions, such as adsorption, precipitation, and fixation, which are significantly affected by soil mineral composition, pH levels, organic matter content, and microbial activity [4,5]. In the soils of the IGP, there is significant variability regarding texture, organic matter, and pH, leading to notable differences in phosphorus availability. In this area, alluvial soils vary from acidic Inceptisols in the eastern IGP to alkaline calcareous soils in the western IGP, with both extremes posing difficulties for P availability. In acidic soils, Fe and Al oxides usually fix P, whereas in calcareous soils, Ca–P precipitation prevails, decreasing the solubility of phosphate ions. As a result, a significant portion of applied fertilizer phosphorus quickly becomes inaccessible to plants, leading to low phosphorus use efficiency, below 20% during the initial season [6].

Phosphate potential (PP) and phosphate ion concentration has been extensively utilized to characterize the level of accessible phosphorus in soil solution [7]. The concentration of

phosphate ions indicates the direct availability of P in the soil solution, whereas phosphate potential considers the soil's buffering capacity and its capability to sustain solution P as it is taken up by plants. Soils that have low phosphate potential cannot sustain sufficient P in solution, leading to significant crop reactions to fertilizer use [7,8]. In contrast, soils with elevated phosphate potential might exhibit reduced responses due to a higher natural P availability. These indices create an important structure for understanding soil P behavior beyond just basic soil test results and assist in clarifying varying crop reactions among different soils [9].

An additional aspect of phosphorus nutrition in wheat is its relationship with micronutrients, especially zinc (Zn). In soils and plants, phosphorus and zinc have an antagonistic interaction referred to as "P-induced Zn deficiency" [10]. The disparity between the supply of macronutrients (like P) and the accessibility of micronutrients (such as Zn) poses a significant challenge to attaining both high yields and nutritional quality in wheat [11].

Earlier research on phosphorus availability in the IGP has mainly concentrated on soil test phosphorus and the responses in crop yields [12]. Nevertheless, these methods frequently do not reflect the dynamic characteristics of soil phosphorus and its relationship with crop needs. Using phosphate ion concentration and phosphate potential as indicators offers a deeper mechanistic insight into P availability. When paired with data on plant uptake and growth, these indices can assist in pinpointing soils that show a strong response to P application compared to those that exhibit a weaker response.

Considering the increasing challenges of sustaining wheat productivity under conditions of nutrient imbalance and environmental stress, a systematic evaluation of phosphorus dynamics in relation to soil properties and wheat response is urgently needed. The Indo-Gangetic Plains provide an ideal setting for this investigation because of their agro ecological diversity, intensive

cropping, and central role in India's food security. In this context, the present study was undertaken (i) To characterize the physico-chemical properties of representative soils from the Indo-Gangetic Plains in relation to phosphorus availability (ii) To quantify phosphate ion concentration and phosphate potential of the soils and assess their variability and (iii) To evaluate the growth, phosphorus uptake, and nutrient interactions of wheat grown on these soils under control and phosphorus-fertilized conditions.

Material and methods

Location and collection of soil samples

The present study requires soil samples with a wide variation in physical and chemical properties. To ensure variation in various physical and chemical properties of soil, including P status, soil samples were collected from 25 locations of Indo Gangetic Plains of India with varying pH and soil physic-chemical properties (Fig 1). The collected soil samples were first air-dried and then ground well. Then it was passed through 2 mm sieve, and a part of the processed soil samples was used for characterization of different initial physico-chemical properties viz., texture, pH, organic carbon, available nitrogen, available phosphorus, available potassium and DTPA extractable zinc (Zn) content using standard analysis protocols, while another part was used for green-house pot experiment.

Fig 1 location map of soil sampling sites in the study region

Phosphate potential

The calculations for phosphate potential (PP) are given below as described by Aslyng [13] :

Phosphate Potential (PP) = 1/2 pCa + pH_2PO_4……………………(i)

1/2 pCa = -1/2 (log C_{Ca} + log f_{Ca})……………………………….(ii)

Where, C_{Ca} = 0.01 M

$$\log f_{Ca} = \frac{0.5\, z^2 \sqrt{\mu}}{1+1.5\sqrt{\mu}} \text{……………………………….(iii)}$$

Where,

z = Valency of ion ; *p* = ionic strength

$$\mu = 1/2 \sum CiZ_{i^2} \text{……………………………….(iv)}$$

Where, C= $H_2PO_4^-$ ion concentration

$pH_2PO_4 = pC_{H_2PO_4} + pf_{H_2PO_4}$ …………………………………………………………(v)

pH_2PO_4 = -log (log C_{H2PO_4} + log $f_{H_2PO_4}$)……………………….(vi)

pC_{H2PO4} =pP + P (H^+/K^{ll} + H^+)………………………………….(vii)

Where,

P = total concentration of phosphorus in the solution

P (H^+/K^{ll} + H^+) = correction factor derived by Aslyng [13] which relates the ratio H_2PO_4/P to pH.

K^{ll} = second ionization constant of H_2PO_4 i.e., $10^{-7.2}$

pP = -log (P concentration)

$$\log f_{H2PO4} = \frac{0.5\, z^2 \sqrt{\mu}}{1+1.5\sqrt{\mu}} \text{………………………………….(viii)}$$

Greenhouse pot experiment

The greenhouse experiment was conducted in pots using all 25 experimental soils to assess the P uptake by plant using wheat (*Triticum aestivum* L) as a test crop. Three kg of soil was poured into plastic pots. A uniform basal dose (150% of the recommended dose) of N, P_2O_5 and K_2O was added to the soil of each pot through urea, di ammonium phosphate and muriate of potash, respectively. All the NPK fertilizers were added in solution form and thoroughly mixed with soil. Each treatment combination was replicated thrice in a completely randomized design using 150 pots. The soil in each pot was then irrigated to field capacity with deionized water and incubated for one week at ambient temperature. Fifteen wheat seeds (variety HD 3086) were sown and after two weeks of sowing, a uniform plant population (five plants pot^{-1}) was maintained in each pot. Pots were watered daily with the required amount of water on weight basis to maintain field capacity. The plants were harvested at the flowering stage, *i.e.*, after 65 days of sowing.

Analysis of plant samples

The harvested above ground plant samples were then washed first under a running tap water, followed by rinsing with dilute HCl (0.001 *N*), distilled water and finally with deionized water. Washed samples were first air-dried. After that, they were kept in hot air oven at 65 ± 2 °C. When the samples attained constant weight, dry biomass yield of the plant samples were recorded and further they were ground in a mechanical grinder made of stainless steel. Ground samples were stored in paper packets for further analysis. Plant samples were digested using a di-acid mixture (HNO_3:$HClO_4$::10:4). 0.5 g ground plant sample was taken in 150 mL conical flask and it was predigested with 10 mL of concentrated HNO_3. Then 5 mL of di-acid mixture was added to it and the flasks were kept on hot plate as described by Piper [14]. After digestion, the

material was cooled, diluted, filtered and the volume was made up to 100 mL. Total P content of the digested samples were determined by vanadomolybdo-phosphoric yellow colour method at 420 nm using a UV-VIS spectrophotometer. Zinc content of the samples was estimated using atomic adsorption spectrophotometer.

Statistical analysis

The data generated were subjected to analysis of variance (ANOVA) technique to know significant difference among treatments. Duncan's Multiple Range Test (DMRT) test was used for multiple comparisons among the treatments at $p < 0.05$ using GRAPES (General R-based Analysis Platform for Experimental Statistics) developed by Kerala Agricultural University (kaugrapes.com).

Results

Initial soil physico-chemical properties

Soil samples selected for the present investigation shows a wide range of variation in physical and chemical properties (Table 1). Texture varied considerably: sandy loam was predominant (S1, S2, S3, S7, S9, S12, S18, S19, S25), followed by sandy clay loam (S5, S8, S16, S20, S22), loam (S4, S13, S17, S21), clay loam (S14, S15, S23, S24), silt loam (S11), and clay (S10). Soil reaction showed marked diversity, ranging from strongly acidic (pH 4.3 in S20, 5.0 in S19) to strongly alkaline (pH 9.1 in S4), suggesting varying degrees of base saturation and exchangeable acidity. Oxidizable organic carbon (OC) levels were generally low, ranging from 0.14% (S5, S12) to 0.90% (S21), reflecting organic matter depletion under intensive cultivation. Available nitrogen ranged from as low as 92 kg/ha (S5) to as high as 347 kg/ha (S25), indicating large

variability in soil fertility status. Phosphorus availability differed widely; the lowest values were 2.48 kg/ha (S6) and 3.45 kg/ha (S22), whereas soils S19 and S20 showed unusually high P availability (65.4 and 33.48 kg/ha, respectively), attributed to soil acidity enhancing solubilization. Potassium availability ranged between 98 kg/ha (S23) and 482 kg/ha (S2). DTPA-extractable Zn showed moderate to wide variation, from deficient levels (0.4 mg/kg in S21, S24) to adequate (2.8 mg/kg in S19). Overall, the data indicate that soils in the region differ significantly in fertility, nutrient reserves, and chemical environment, which directly influences nutrient dynamics and crop responses.

Table 1 Initial physico-chemical properties of selected soils of Indo Gangetic Plains

S.No.	Sand (%)	Silt (%)	Clay (%)	Texture	pH	OC (%)	Avail. N (kg/ha)	Avail. P (kg/ha)	Avail. K (kg/ha)	DTPA-Zn (mg/kg)
S1	77.68	10	12.32	Sandy loam	6.7	0.31	301	35.12	291	1.1
S2	74.38	15	10.62	Sandy loam	8.1	0.49	121	27.49	482	0.7
S3	75.68	12	12.32	Sandy loam	8.6	0.20	100	10.59	149	1.2
S4	43.68	34	22.32	Loam	9.1	0.32	171	4.50	183	1.2
S5	58.4	18	23.6	Sandy clay loam	8.5	0.14	92	18.24	308	0.7
S6	66.0	16	18	Sandy loam	8.6	0.67	205	2.48	350	1.0
S7	71.68	14	14.32	Sandy loam	8.1	0.49	171	5.18	229	2.1
S8	53.7	21.7	24.6	Sandy clay loam	8.3	0.20	151	10.19	352	1.0
S9	53.68	28	18.32	Sandy loam	7.9	0.36	180	6.33	100	1.1
S10	18.4	38	43.6	Clay	7.4	0.16	151	14.16	189	1.7
S11	25.5	66.5	8.0	Silt loam	7.6	0.40	167	13.38	150	0.9
S12	56.32	27.52	16.16	Sandy Loam	8.1	0.14	117	10.63	153	1.1

S13	30.4	46	23.6	Loam	7.7	0.36	243	13.48	132	1.1
S14	34.4	38	27.6	Clay loam	7.9	0.19	243	18.69	327	0.9
S15	40.88	29	30.12	Clay loam	7.2	0.48	197	14.22	145	0.6
S16	55.6	15.5	28.9	Sandy Clay loam	7.2	0.42	243	11.71	126	0.9
S17	48.4	34	17.6	Loam	8.4	0.52	171	30.28	177	1.4
S18	55	35	10	Sandy loam	8.6	0.15	159	9.38	149	0.9
S19	64.4	24	11.6	Sandy loam	5.0	0.46	230	65.40*	160	2.8
S20	63.5	17.3	23.2	Sandy clay loam	4.3	0.69	280	33.48*	372	0.9
S21	37	40	23	Loam	7.9	0.90	301	3.51	155	0.4
S22	56	23	21	Sandy clay loam	6.7	0.47	243	3.45	121	0.9
S23	22.4	50	27.6	Clay loam	6.4	0.53	230	29.23	98	1.1
S24	25.68	44	30.32	Clay loam	7.0	0.30	234	11.60	220	0.4
S25	64.4	20	15.6	Sandy loam	6.3	0.54	347	38.68	346	0.9

*Bray’s P-1 Phosphorus

S1-S25: location of sampling

Phosphate potential

Marked variability was observed in phosphate ion concentration and phosphate potential among the soils (Table 2). Extractable $H_2PO_4^-$ ranged from as low as 390 µM in S19 to an extremely high 3901 µM in the same soil under acidic conditions, emphasizing the strong influence of soil pH on phosphate solubility. Acidic soils such as S19 and S20 had markedly higher $H_2PO_4^-$ concentrations (3901 and 1832 µM, respectively), while alkaline soils like S6, S9, and S10 showed much lower values (442–563 µM). The phosphate potential (PP) varied from 3.55 (S19)

to 4.55 (S13 and S16), indicating contrasting phosphate buffering capacities across soils. pfH_2PO_4 values were highest in S19 (0.0207) and S20 (0.0125), reflecting greater phosphate intensity in acidic soils, while lowest values (0.007–0.0073) were recorded in alkaline sandy loam soils (S6, S12, S16). Soils S17 and S2 also showed moderately high $H_2PO_4^-$ values (1777 and 1560 µM, respectively), while soils S4 and S9 recorded lower levels, suggesting stronger P fixation tendencies. The data demonstrate that soil pH, texture, and organic matter collectively influence phosphate intensity and buffering. Acid soils are characterized by higher P solubility but lower buffering capacity, whereas alkaline soils show stronger buffering, limiting immediate P availability. This highlights the complexity of phosphorus dynamics across contrasting soil environments of the Indo-Gangetic Plains, where both inherent properties and management interventions decide nutrient supply to crops.

Table 2 Phosphate ion concentration and phosphate potential of the selected soils of Indo Gangetic plains

S.No	pH ($CaCl_2$) extractable)	$H_2PO_4^-$ (M) (*10-6)	$pH_2PO_4^-$	CF	pfH_2PO_4	Phosphate potential (PP)
S1	6.35	874cd	3.06def	0.0008	0.0101	4.20def
S2	7.51	1560bc	2.81gh	0.0006	0.0134	3.95gh
S3	7.58	762cd	3.13bcdef	0.0002	0.0094	4.27bcdef
S4	7.46	532d	3.28abcd	0.0002	0.008	4.42abcd
S5	7.28	1099bcd	2.96fg	0.0005	0.0113	4.10fg
S6	7.36	453d	3.35ab	0.0002	0.0073	4.49ab
S7	7.14	1244bcd	2.91fgh	0.0007	0.012	4.05fgh
S8	7.13	795cd	3.10cdef	0.0004	0.0097	4.24cdef
S9	7.1	476d	3.33abc	0.0003	0.0075	4.47abc
S10	6.7	472d	3.33abc	0.0004	0.0075	4.47abc
S11	6.77	819cd	3.09def	0.0006	0.0098	4.23def
S12	7.09	442d	3.36a	0.0003	0.0073	4.50a
S13	6.96	397d	3.41a	0.0003	0.0069	4.55a
S14	7.18	1021bcd	2.99efg	0.0005	0.0109	4.13efg
S15	6.54	563d	3.26abcd	0.0005	0.0081	4.40abcd

S16	6.52	409d	3.39a	0.0003	0.007	4.53a
S17	7.33	1777b	2.75h	0.0008	0.0143	3.89h
S18	7.41	587d	3.23abcd	0.0002	0.0083	4.37abcd
S19	4.75	3901a	2.41i	0.0039	0.0207	3.55i
S20	4.75	1832b	2.99fg	0.0018	0.0125	4.13fg
S21	6.92	560d	3.25abcd	0.0004	0.0082	4.39abcd
S22	6.66	620d	3.21abcde	0.0005	0.0086	4.35abcde
S23	5.94	560d	3.25abcd	0.0005	0.0082	4.39abcd
S24	6.92	434d	3.36a	0.0003	0.0072	4.50a
S25	5.93	593d	3.23abcd	0.0006	0.0084	4.37abcd

S1-S25: location of sampling; Values followed by different superscript capital letters in a column and mean values followed by different capital letters are significantly different ($P<0.05$).

Dry weight and P uptake

Wheat responded variably to phosphorus application across soils, highlighting the contrasting nutrient supply capacities (Table 3). Dry weight under control ranged from just 4.07 g/pot (S19) to 14.9 g/pot (S16), with marked improvement under P fertilization, reaching up to 16.4 g/pot (S14) and 16.1 g/pot (S19, S20, S24). The relative increase in biomass due to P was particularly observed in low-P soils such as S19 (from 4.07 to 16.1 g/pot) and S24 (from 5.30 to 15.9 g/pot), indicating high fertilizer responsiveness. Phosphorus concentration in wheat plants also increased with P application, from a minimum of 0.37 mg/g (S13, control) to 3.57 mg/g (S23, fertilized). P uptake followed the same trend, with low uptake in controls (5.20 mg/pot in S13) and significant enhancement under fertilization, peaking at 54.7 mg/pot (S19) and 49.2 mg/pot (S15). Across soils, the magnitude of increase in P uptake varied widely, with some soils (S13, S24) showing more than fivefold increases. In general, soils with low inherent available P (S19, S23, S24) responded strongly to fertilization, while those with moderate P (S7, S8, S17) showed relatively less improvement. The data reveal that phosphorus application consistently improved

plant growth and nutrient acquisition, though the efficiency of uptake was soil-specific, underlining the role of inherent soil fertility and P dynamics in influencing crop response.

Cumulative data shows clear positive effects of P fertilization on wheat growth and nutrient status (Fig 2). Across soils, P application significantly increased shoot dry matter, tissue P concentration, and total uptake compared to unfertilized controls. While the degree of response varied, low-P soils showed the largest relative improvements. The data shows that native soil P was insufficient in most soils to support optimal wheat growth. Fertilization not only enhanced biomass but also improved nutrient concentration, indicating better physiological utilization of phosphorus. The trend underscores the importance of P supplementation in maximizing crop productivity in the Indo-Gangetic Plains.

Table 3 Dry weight, P concentration and uptake by wheat in selected soils of Indo Gangetic Plains

Treatment	Dry weight (g/pot)		P Concentration (mg/g)		P Uptake (mg/pot)	
	Control	100 % P	Control	100 % P	Control	100 % P
S1	10.9±0.9	11.7±1.2	2.10±0.24	2.60±0.04	23.0±4.4	30.5±3.3
S2	8.13±0.7	13.5±1.2	0.88±0.04	2.79±0.06	7.20±0.4	37.5±3.0
S3	12.7±1.1	14.4±1.1	1.62±0.21	1.75±0.06	20.6±4.3	25.3±2.4
S4	10.6±0.8	13.8±1.9	1.23±0.04	1.49±0.10	13.1±1.1	20.4±1.4
S5	10.7±0.4	13.4±1.1	2.04±0.15	2.54±0.07	21.9±1.4	34.0±2.2
S6	7.93±0.5	12.3±2.1	0.93±0.03	1.69±0.38	7.40±0.7	20.3±1.3
S7	12.8±0.6	14.6±1.5	2.36±0.14	2.58±0.03	30.2±3.0	37.7±4.4
S8	14.1±0.7	14.7±2.1	1.63±0.17	2.15±0.40	22.9±1.7	31.9±9.4
S9	10.7±0.9	12.0±0.6	0.99±0.12	2.73±0.37	10.6±1.9	32.9±5.8
S10	7.27±0.8	13.9±1.7	1.02±0.08	1.24±0.10	7.50±1.3	17.2±1.9
S11	6.03±0.9	14.6±2.4	1.07±0.12	1.43±0.05	6.40±0.4	20.8±3.1
S12	10.6±1.6	14.8±0.3	0.65±0.08	2.43±0.08	6.80±0.6	36.0±2.0
S13	14.2±0.7	14.2±1.1	0.37±0.09	2.90±0.18	5.20±1.2	41.4±5.3
S14	10.3±0.8	16.4±1.3	2.02±0.16	2.67±0.13	20.7±0.4	43.7±5.1
S15	13.3±0.3	14.7±0.1	1.41±0.14	3.34±0.15	18.8±2.0	49.2±1.9

S16	14.9±0.5	15.8±0.5	0.64±0.12	1.54±0.07	9.60±2.0	24.3±0.5
S17	14.3±0.7	14.7±0.3	2.63±0.22	2.93±0.04	37.6±5.0	43.2±1.3
S18	11.0±0.6	15.0±0.3	1.75±0.04	1.83±0.03	19.2±0.6	27.3±0.5
S19	4.07±0.5	16.1±1.7	3.08±0.12	3.39±0.25	12.6±2.0	54.7±9.9
S20	14.4±0.7	16.1±1.4	1.07±0.21	1.66±0.06	15.5±3.7	26.8±2.8
S21	11.5±1.3	13.8±1.7	1.48±0.21	1.87±0.07	17.2±4.4	25.8±2.3
S22	11.0±0.3	11.5±0.6	1.81±0.27	2.46±0.02	19.9±2.6	28.2±1.4
S23	10.7±1.4	13.5±1.4	1.27±0.25	3.57±0.17	13.5±1.2	48.1±2.8
S24	5.30±0.3	15.9±2.0	1.67±0.05	2.33±0.09	8.80±0.1	37.2±5.9
S25	12.0±0.4	15.6±1.6	1.47±0.27	1.67±0.04	17.6±2.9	26.3±2.1
CV	7.43	9.87	10.81	7.09	15.5	12.4
LSD	1.31	2.31	0.26	0.27	4.00	6.66
SE(m)	0.46	0.81	0.09	0.09	1.41	2.35

S1-S25: location of sampling

Fig 2. Cumulative P uptake, concentration, and dry weight

Zn×P interaction

Zinc concentration in wheat straw showed significant response under P application. In the control, Zn concentration ranged between 5.70 mg/kg (S24) and 18.3 mg/kg (S15). With P fertilization, values shifted widely, dropping drastically in soils such as S25 (0.92 mg/kg) and S6 (4.34 mg/kg), while increasing in S15 (22.7 mg/kg). This demonstrates a complex interaction where P fertilization often diluted or antagonized Zn uptake. The P/Zn ratio showed even more variation. In controls, ratios ranged from as low as 24 (S13) to as high as 293 (S24), while with P fertilization they increased sharply, particularly in S25 (1818), indicating severe nutrient imbalance. In contrast, in soils with better Zn availability (S15, S17, S19), ratios were relatively stable (147–209). Overall, P application enhanced P nutrition but often at the cost of Zn uptake, leading to elevated P/Zn ratios. This imbalance poses a risk of inducing Zn deficiency in wheat

grown on soils with marginal Zn reserves, emphasizing the need for balanced nutrient management strategies.

Table 4 Zinc (Zn) concentration and P/Zn ratio in wheat straw in selected soils of Indo Gangetic Plains

Treatment	Zn Concentration (mg/kg)		P/Zn ratio	
	Control	100 % P	Control	100 % P
S1	7.88±0.4	7.74±0.4	267±37	336±23
S2	8.20±0.3	6.94±0.3	108±10	402±11
S3	10.7±0.2	10.1±0.2	152±22	174±5
S4	17.9±1.7	17.2±1.6	69±9	87±4
S5	14.1±0.8	13.6±0.8	146±15	186±15
S6	7.52±0.2	4.34±0.1	123±7	390±79
S7	9.94±0.5	7.74±0.4	237±6	334±14
S8	9.66±0.8	9.14±0.7	170±26	239±64
S9	11.5±0.5	9.86±0.4	86±9	276±29
S10	11.7±0.6	7.28±0.4	88±10	171±21
S11	15.2±0.5	14.1±0.5	70±11	101±7
S12	14.9±0.4	13.6±0.4	43±4	179±6
S13	15.2±0.8	8.32±0.5	24±5	349±12
S14	9.94±0.5	7.86±0.4	203±20	340±20
S15	18.3±1.0	22.7±1.2	77±12	147±14
S16	11.5±0.7	14.4±0.9	56±11	107±3
S17	13.6±0.5	17.5±0.6	192±9	168±8
S18	8.20±0.2	6.06±0.2	214±9	302±6
S19	17.6±0.4	16.2±0.3	175±10	209±12
S20	7.86±0.4	6.72±0.4	136±30	248±19
S21	8.44±0.5	9.94±0.6	176±27	189±13
S22	7.98±0.3	9.48±0.3	226±26	260±9
S23	15.2±0.2	14.9±0.1	84±17	240±13
S24	5.70±0.3	12.6±0.7	293±12	186±7
S25	6.72±0.2	0.92±0.1	219±34	1818±115
CV	5.31	5.60	12.4	11.2
LSD	0.99	0.99	29.6	54.4
SE(m)	0.35	0.35	10.4	19.2

S1-S25: location of sampling

Correlation

The correlation analysis revealed contrasting patterns under control and fertilized conditions. In fertilized pots, strong positive correlations were observed between P uptake and dry matter, as well as between uptake and concentration, indicating that improved P availability drives growth and nutrient accumulation simultaneously. In contrast, under control conditions, correlations were weaker, suggesting that limited P supply constrained the relationship between growth and nutrient status. The results highlight the central role of phosphorus in determining productivity and nutrient dynamics in these soils.

Figure 3. Correlation matrix of control vs. P fertilized pots

Discussion

The present study demonstrated considerable variability in the physico-chemical properties of soils from the Indo-Gangetic Plains (IGP), which in turn strongly influenced phosphorus (P) availability, wheat growth response, and the interaction with zinc (Zn). Across the 25 soils evaluated, differences in soil pH, texture, organic carbon, and baseline nutrient content were large, reflecting the well-known heterogeneity of IGP soils under intensive cultivation. Such variability has profound implications for nutrient management and crop productivity.

Soil fertility status at the outset (Table 1) indicated that organic carbon was consistently low, and that available nitrogen, phosphorus, and potassium were unevenly distributed. This is consistent with earlier studies showing the depletion of organic matter and imbalances in macro- and micronutrients in intensively cropped IGP systems [15-17]. The large spread in soil reaction (pH

4.3 to 9.1) reflects both inherent parent material and long-term management, and directly conditioned P dynamics. Acidic soils (e.g., S19, S20) exhibited unusually high extractable P, likely due to enhanced solubilization under low pH, while alkaline soils showed restricted P solubility due to precipitation with Ca. These findings align with previous reports indicating that pH is the single most important driver of phosphate solubility and fixation in IGP soils [18-20].

The analysis of phosphate ion concentrations and phosphate potential (Table 2) further reinforced this observation. Acidic soils presented higher phosphate intensity but weaker buffering capacity, whereas alkaline soils exhibited strong buffering but lower intensity. Such results highlight the inherent trade-off between solubility and buffering, which has been documented in other Indian soils [18,21]. For crop management, this means that identical rates of P fertilizer will behave differently depending on soil buffering: in low-buffer soils, applied P remains more available but is prone to leaching, while in high-buffer soils, larger applications may be required to overcome fixation. The pronounced variability in pfH_2PO_4 values also suggests that soils differ in their ability to maintain phosphate concentration against plant uptake, which may explain subsequent differences in crop response.

Wheat grown in pots without P fertilization had generally low biomass and nutrient uptake, confirming that native soil P was insufficient for optimal growth. Addition of 100% P fertilizer significantly improved dry matter, tissue P concentration, and total uptake (Table 3). The relative gain was most pronounced in soils with very low baseline P, such as S19 and S24, where biomass tripled or quadrupled after fertilization. This is in agreement with earlier reports that the majority of IGP soils are highly responsive to P fertilization, particularly those under rice–wheat sequences that have experienced nutrient mining [19,22,23]. The cumulative response trends (Figure 2) clearly demonstrated that P application was essential to unlock yield potential, and the

correlation analysis (Figure 3) highlighted that under P sufficiency, plant growth, P concentration, and uptake were tightly coupled. In contrast, under deficiency, these relationships weakened, illustrating that P availability is the primary factor regulating the coordination between growth and nutrient accumulation.

An important dimension of the study was the antagonistic effect of P fertilization on Zn nutrition (Table 4). In many soils, P application led to reduced Zn concentration in wheat straw and a substantial increase in the P/Zn ratio. This was especially severe in soils with low baseline Zn, such as S25, where the ratio rose above 1,800. Such imbalances are of agronomic and nutritional concern, since high P/Zn ratios in plants are associated with hidden Zn deficiencies that may impair yield and reduce grain nutritional quality [21,24,25]. The phenomenon of P-induced Zn deficiency has been widely reported and attributed to several mechanisms: precipitation of Zn as insoluble phosphates, competitive inhibition of uptake, suppression of Zn-mobilizing mycorrhizal associations, and dilution effects due to increased biomass [25-27]. Our findings provide empirical evidence of this interaction under IGP soil conditions. Conversely, in soils with adequate Zn reserves (e.g., S15, S17), the negative impact of P was less pronounced, suggesting that Zn sufficiency buffers against P-induced stress.

These results emphasize the need for integrated P and Zn management. Previous field studies in wheat and maize have shown that combined P and Zn fertilization not only improves yield but also alleviates nutrient antagonism [5,22,28]. Co-application strategies, including Zn-enriched superphosphate or blended fertilizers, have been reported to improve nutrient use efficiency and balance P/Zn ratios [26,29]. The findings here suggest that in P-deficient yet Zn-marginal soils, applying P without Zn could exacerbate imbalances. Hence, nutrient recommendations in the IGP should avoid blanket prescriptions and instead incorporate soil test data for both

macronutrients and micronutrients. The heterogeneity observed across the sampled soils supports the argument for site-specific nutrient management.

Beyond chemical fertilizers, soil organic carbon emerges as a critical factor. Across the sampled soils, organic matter was consistently low, which not only reduces nutrient retention but also limits biological processes such as mycorrhizal colonization. Conservation agriculture practices that improve organic matter levels have been shown to enhance nutrient cycling and moderate negative P–Zn interactions [17,19]. Incorporating residues, green manures, or compost could improve both P and Zn availability while enhancing soil structure and microbial health. Building organic matter would also improve phosphate buffering and reduce the volatility of nutrient responses. While P fertilization remains essential to sustain wheat productivity, the concurrent risk of Zn deficiency cannot be ignored. This is particularly relevant in the context of human nutrition, as Zn deficiency in crops translates into dietary deficiencies in populations relying heavily on wheat-based diets [21,25]. Thus, agronomic interventions addressing P–Zn interactions contribute not only to productivity but also to nutritional security.

Conclusion

This study demonstrated that soils of the Indo-Gangetic Plains (IGP) are highly heterogeneous in their physico-chemical properties, leading to marked differences in phosphorus (P) availability, buffering capacity, and crop responsiveness. Soil pH emerged as a key driver of P dynamics, with acidic soils showing higher phosphate intensity but weaker buffering, while alkaline soils exhibited lower intensity but stronger buffering. Wheat grown in these soils responded strongly to P fertilization, particularly in low-P soils, with significant improvements in biomass, tissue P concentration, and uptake. However, P addition frequently depressed zinc (Zn) concentration in

wheat straw, increasing the P/Zn ratio and indicating a risk of nutrient imbalance. The antagonistic interaction between P and Zn was most severe in soils with low baseline Zn availability, underscoring the potential for P-induced Zn deficiency under fertilization regimes that ignore micronutrient management.

These findings highlight the need for soil-specific and balanced nutrient management strategies in the IGP. Blanket recommendations of P fertilizers are unlikely to be effective and may exacerbate micronutrient deficiencies. Instead, integrated P and Zn fertilization, informed by soil testing, should be prioritized to maximize yield while safeguarding nutritional quality. Conservation agriculture practices and organic matter management could further improve nutrient buffering and enhance the biological processes that support both P and Zn uptake.

By integrating soil fertility mapping, site-specific nutrient management, and balanced fertilization strategies, sustainable wheat production in the IGP can be achieved. Such approaches will not only improve productivity but also contribute to addressing widespread micronutrient malnutrition in human populations dependent on wheat-based diets. Ultimately, managing the interaction between P and Zn is essential to reconcile crop yield goals with soil health and nutritional security in one of the world's most intensively cultivated agricultural regions.

Acknowledgement

This work was carried out to fulfill the requirement of PhD programme in the division of Soil Science and Agricultural Chemistry at ICAR- Indian Agricultural Research Institute, New Delhi. The authors are thankful to the Director, ICAR-IARI, Professor and faculty of the soil science

division for providing laboratory facilities for conducting the experiment and analysis of soil and plant samples.

Declaration on conflict of interest

The authors affirm that there are no conflicts of interest to disclose.

Author contributions

Conceptualization: K. K. Rao, Mandira Barman, Debarup Das, Vinod Kumar Sharma, T.K. Das

Data curation: K.K. Rao, Mandira Barman, Debrup Ghosh, H. Krishna

Funding acquisition: H. Krishna

Investigation: K. K. Rao,

Supervision: Mandira Barman, Debarup Das, Vinod Kumar Sharma, T.K. Das

Writing-original draft: K. K. Rao,

Writing-review 7 editing: K. K. Rao, Mandira Barman, Debarup Das, Vinod Kumar Sharma, T.K. Das, Debrup ghosh, N. Krishna

References

1. McDonald AJ, Keil A, Srivastava A, Craufurd P, Kishore A, Kumar V, Paudel G, Singh S, Singh AK, Sohane RK, Malik RK. Time management governs climate resilience and productivity in the coupled rice–wheat cropping systems of eastern India. Nature Food. 2022 Jul;3(7):542-51.
2. Ramdas S, Singh R, Sharma I. Exploring the performance of wheat production in India. Journal of Cereal Research. 2013;4(2).
3. Koshal AK. Changing current scenario of rice-wheat system in indo-gangetic plain region of India. International Journal of Scientific and Research Publications. 2014;4(3):1-3.
4. Hu D, Zhang J, Yang Y, Yu D, Zhang H, Zhang D. Molecular mechanisms underlying plant responses to low phosphate stress and potential applications in crop improvement. New Crops. 2025 Jan 4:100064.

5. George TS, Hinsinger P, Turner BL. Phosphorus in soils and plants–facing phosphorus scarcity. Plant and Soil. 2016 Apr;401(1):1-6.
6. Raychaudhuri M, Kundu DK, Kumar A, Mandal KG, Raychaudhuri S, Kar G, Bhattacharyya T, Sarkar D, Pal DK, Mandal DK, Prasad J. Soil physical quality of the Indo-Gangetic Plains and black soil region. Current Science. 2014 Nov 10:1440-51.
7. Syers JK, Johnston AE, Curtin D. Efficiency of soil and fertilizer phosphorus use. FAO Fertilizer and plant nutrition bulletin. 2008;18(108):5-0.
8. Shirvani M, Shariatmadari H, Kalbasi M. Phosphorus buffering capacity indices as related to soil properties and plant uptake. Journal of plant nutrition. 2005 Mar 16;28(3):537-50.
9. Chen X, Yan X, Wang M, Cai Y, Weng X, Su D, Guo J, Wang W, Hou Y, Ye D, Zhang S. Long-term excessive phosphorus fertilization alters soil phosphorus fractions in the acidic soil of pomelo orchards. Soil and Tillage Research. 2022 Jan 1;215:105214.
10. Zhang W, Zhang W, Wang X, Liu D, Zou C, Chen X. Quantitative evaluation of the grain zinc in cereal crops caused by phosphorus fertilization. A meta-analysis. Agronomy for Sustainable Development. 2021 Feb;41(1):6.
11. Miner GL, Delgado JA, Ippolito JA, Johnson JJ, Kluth DL, Stewart CE. Wheat grain micronutrients and relationships with yield and protein in the US Central Great Plains. Field Crops Research. 2022 Apr 1;279:108453.
12. Jiang B, Jianlin SH, Minghong SU, Yajun HU, Jiang W, Juan WA, Yong LI, Jinshui WU. Soil phosphorus availability and rice phosphorus uptake in paddy fields under various agronomic practices. Pedosphere. 2021 Feb 1;31(1):103-15.

13. Aslyng HC. Phosphate potential and phosphate status of soils. Acta Agriculturae Scandinavica. 1964 Jan 1;14(4):261-85.

14. Piper, CS. (1967). *Soil and plant analysis*. Adelaide, Australia: University of Adelaide.

15. Kumar A, Bairwa M, Meena SK, Singh SK, Sinha SK, Rana L, Singh YV, Kumar A, Singh H, Paswan S. Nutrient index value as a tool for evaluating soil fertility in diverse agroecosystems of Indo-Gangetic plains of India. AATCC review. 2025 Jan 25;13(2):415-27.

16. Kumar A, Bairwa M, Meena SK, Singh SK, Sinha SK, Rana L, Singh YV, Kumar A, Singh H, Paswan S. Comparative Analysis of Macronutrient, Soil Sulphur Availability and Micronutrient Dynamics across Diverse Land Use Types of Indo-Gangetic plains of India. AATCC review. 2025 Feb 23;13(01):467-72.

17. Rao KK, Samal SK, Poonia SP, Kumar R, Mishra JS, Bhatt BP, Dwivedi SK, Mondal S, Choubey AK, Kumar S, Kumar M. Conservation agriculture improves soil physical properties and crop productivity: a long-term study in middle Indo-Gangetic Plains of India. Soil Research. 2021 Oct 26;60(6):442-54.

18. Dutta A, Hazra KK, Nath CP, Kumar N, Singh U, Singh SS, Praharaj CS. Long-term impact of crop diversification and nutrient management on soil phosphorus pools in Indo-Gangetic Plain. Communications in Soil Science and Plant Analysis. 2024 Oct 10;55(18):2667-86.

19. Reddy KS, Parihar CM, Panneerselvam P, Sarkar A, Nayak HS, Patra K, Sena DR, Reddy GS, Sinha A, Bharadwaj S, Kumar S. Eco-optimizing rice-wheat system of Eastern Indo-Gangetic plains of India through resource conservation technologies:

insights from field experiments and modeling. Frontiers in Sustainable Food Systems. 2025 Jan 31;9:1499425..

20. Hazra KK, Swain DK, Singh SS, Nath CP. System-based phosphorus management in non-flooded rice-lentil rotation in tropical alkaline soil on Gangetic plain: Crop productivity, phosphorus use efficiency and budgeting. Field Crops Research. 2024 Jul 1;315:109438.
21. Yu BG, Chen XX, Cao WQ, Liu YM, Zou CQ. Responses in zinc uptake of different mycorrhizal and non-mycorrhizal crops to varied levels of phosphorus and zinc applications. Frontiers in Plant Science. 2020 Dec 3;11:606472.
22. Dhaliwal SS, Sharma V, Shukla AK, Behera SK, Verma V, Kaur L, Verma G. Optimization of phosphorus (P) induced zinc (Zn) deficiency in low and high P soils and monitoring their effect on productivity, Zn and P uptake by wheat (Triticum aestivum L.). Journal of Plant Nutrition. 2023 Apr 3;46(6):917-28.
23. Ali M, Ahmed I, Zia MH, Abbas S, Sultan T, Sharif M. Enhancing wheat yield and zinc biofortification through synergistic action of potent zinc-solubilizing bacteria and zinc sulfate in calcareous soil. Agricultural Research. 2025 Mar;14(1):159-70.
24. Nguyen TD, Cavagnaro TR, Watts-Williams SJ. The effects of soil phosphorus and zinc availability on plant responses to mycorrhizal fungi: a physiological and molecular assessment. Scientific reports. 2019 Oct 16;9(1):14880.
25. Cakmak I, Marschner H. Mechanism of phosphorus-induced zinc deficiency in cotton. III. Changes in physiological availability of zinc in plants Is mail. Physiologia Plantarum. 1987 May;70(1):13-20.
26. CropNutrition. Don’t forget zinc when applying phosphorus. Nutrien Ag Solutions. 2023.

27. Marschner H, editor. Marschner's mineral nutrition of higher plants. Academic press; 2011 Aug 8.

28. Sánchez-Rodríguez AR, Rey MD, Nechate-Drif H, Castillejo MÁ, Jorrín-Novo JV, Torrent J, Del Campillo MC, Sacristán D. Combining P and Zn fertilization to enhance yield and grain quality in maize grown on Mediterranean soils. Scientific reports. 2021 Apr 1;11(1):7427.

29. Mortvedt JJ. Micronutrient fertilizer technology. Micronutrients in agriculture. 1991 Jan 1;4:523-48.

Figures Click here to access/download;Figure;Graphs.docx

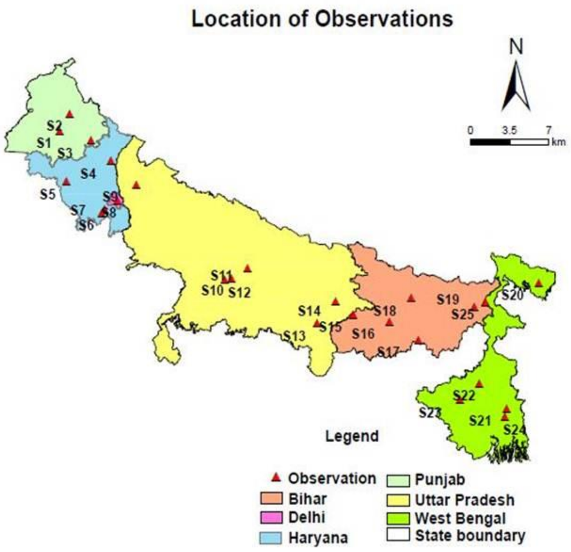

Sample	Sampling location	latitude	longitude
S1	Ludhiana-1	30.5359	75.4741
S2	Ludhiana-2	31.000954	75.770886
S3	Patiala	30.268217	76.389673
S4	Karnal	29.711335	76.965604
S5	Hisar	29.156002	75.67911
S6	Rewari	28.302433	76.697033
S7	Gurugram	28.396131	76.777702
S8	Delhi	28.640848	77.154744
S9	Modipuram	29.078057	77.705439
S10	Lucknow-1	26.5336	80.4657
S11	Lucknow-2	26.804357	80.931392
S12	Kanpur	26.494213	80.269003
S13	Varanasi	25.303994	82.949708
S14	Mau	25.897735	83.487178
S15	Buxar	25.5472	83.991111
S16	Patna	25.3531	85.0507
S17	Nawada	24.849237	85.888289
S18	Samastipur	25.983333	85.683333
S19	Purnea	25.736767	87.509639
S20	Coochbehar	26.402771	89.391102
S21	Kalyani	22.986788	88.454095
S22	Viswabharti	23.669166	87.657941
S23	Kolkata	23.232996	87.096314
S24	Barrackpore	22.759957	88.40163
S25	Uttar Dinajpur	25.883312	87.833562

Fig 1 location map of soil sampling sites in the study region

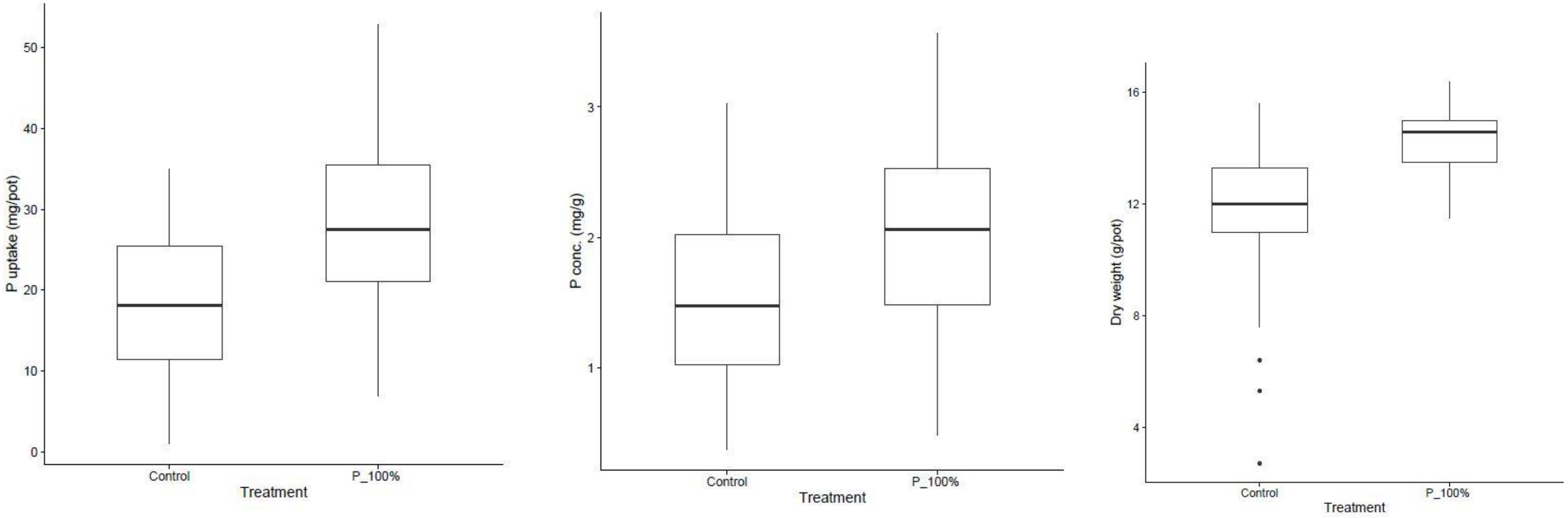

Fig 2 Cumulative P uptake, concentration and dry weight in control and 100 % P treatments

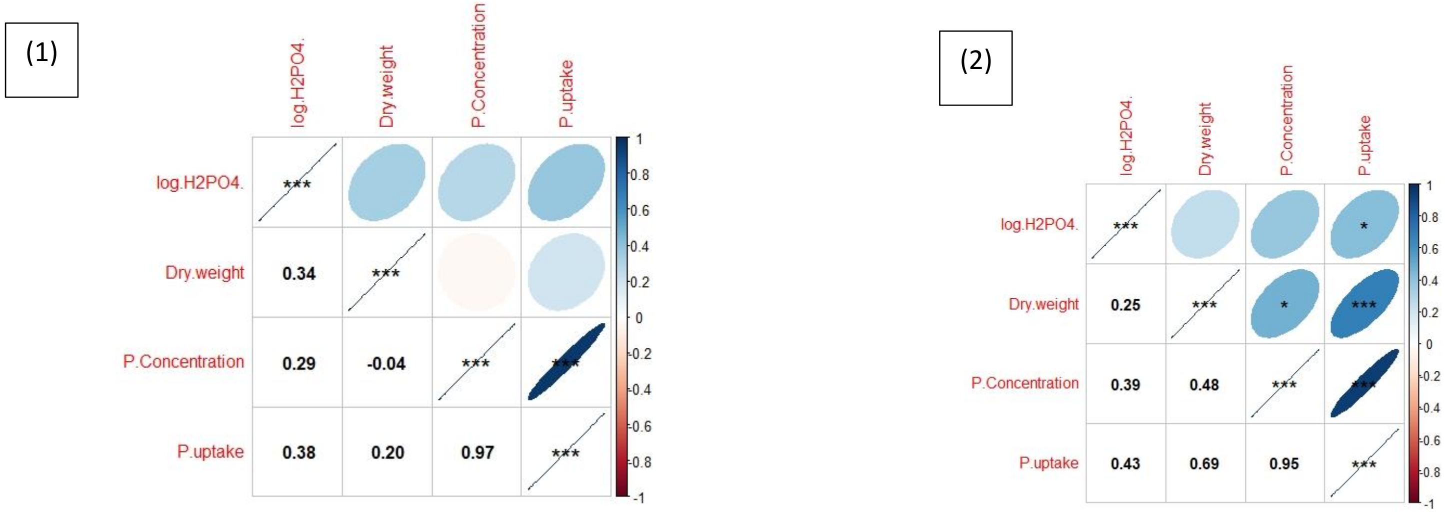

Fig 3 Correlation matrix of (1)control (2) 100 % P applied pots
