# Supplementary material for: Understanding phosphorus dynamics and uptake in diverse soils of Indo-Gangetic Plains of India
Source: PLoS One. 2025 Dec 30;20(12):e0339034. doi: 10.1371/journal.pone.0339034 (PMC12752939; doi:10.1371/journal.pone.0339034)
Supplement: Attachment — Submitted filename: Comments PLOSONE.docx [file pone.0339034.s002.docx]

**Comments**

This manuscript addresses an important topic concerning phosphorus (P) dynamics and uptake in the Indo-Gangetic Plains (IGP), a region of high agronomic significance. The study provides valuable insights into the relationship between soil physico-chemical properties, P availability, and nutrient interactions, particularly the antagonism between P and Zn in wheat. The topic aligns well with the scope of *PLOS ONE*, emphasizing soil fertility, nutrient efficiency, and sustainable crop production.

However, while the study is scientifically sound and the data is extensive, the MS need some minor revisions:

**Title and Abstract**

- The title is appropriate; however, please correct the spelling of *“Phopshorus”* to *“Phosphorus”* in the short title.
- In the abstract, the sentence *“excessive P application altered Zn concentration”* may be rephrased as *“P fertilization altered Zn concentration”* to avoid implying over-application.

**Introduction**

- The introduction is well written and contextually relevant. Ensure consistent use of “phosphorus (P)” after its first occurrence.

**Results**

- In the Tables, please ensure consistent significant figure formatting for numeric data (e.g., 2 decimal places or as appropriate).

**Discussion**

- The discussion effectively relates soil variability and P dynamics and well written while focusing on the most relevant findings.

**Conclusion**

- The conclusion is comprehensive. You may consider a concise final sentence emphasizing the applicability of findings to site-specific nutrient management and nutritional quality improvement.

**Formatting and References**

- Check consistency in reference formatting (especially spacing and punctuation between author initials).
- Ensure that all abbreviations (e.g., OC, DTPA) are defined at first use.
- Correct minor typographical errors (e.g., inconsistent capitalization, spacing before parentheses).

**Some specific suggestions:**

- Line 92: Check grammar of this sentence.
- Line 95: Spelling mistake. Need Correction.
- Line 101: Need Capitalization.
- Line 109: this symbol (p) is not in above equation.
- Line 113, 114, 120: Write properly by using subscripts.
- Line 125: Combine it as diammonium.
- Line 127: Treatment combinations are not defined clearly, neither in a table or in-text.
- Table 1: Maintain the uniformity of digits after decimal through the table. Write expanded form of ‘Avail.’ in the footnote of the table.
- Table 2: Write expanded form of ‘CF’ and other variables in footnote of the table.
- Table 3 &4: Add expanded form and details regarding no. of observations, level of significance, etc. about CV, LSD, and SE(m) terms in table footnote.

**Overall Assessment**

The manuscript is well-structured, data-rich, and scientifically sound. Only minor editorial and formatting improvements are needed. The findings have clear implications for nutrient management in the Indo-Gangetic Plains and merit publication after these small revisions.
