# Supplementary material for: Understanding phosphorus dynamics and uptake in diverse soils of Indo-Gangetic Plains of India
Source: PLoS One. 2025 Dec 30;20(12):e0339034. doi: 10.1371/journal.pone.0339034 (PMC12752939; doi:10.1371/journal.pone.0339034)
Supplement: Attachment — Submitted filename: PLOS_ONE_Reviewer_Report.docx [file pone.0339034.s004.docx]

The manuscript 'Understanding phosphorus dynamics and uptake in diverse soils of Indo-Gangetic Plains of India' presents novel insights into phosphorus dynamics and nutrient interactions in wheat under diverse soil conditions. The work is generally sound and within the scope of PLOS ONE. The manuscript is well written and structured logically. The objectives are clear and align with the presented results. However, a few editorial and methodological clarifications are required. Figures and tables could benefit from consistent formatting, and certain results need quantitative support. Key issues include unclear fertilizer protocols, limited explanation of sampling design and interpretation is largely descriptive.

### Line-by-Line Comments

Lines3: Correct "Phopshorus" to "Phosphorus" in the short title.

Lines 36-37: Add "wheat" and "zinc deficiency" for better discoverability.

Lines 20–35: Abstract: Condense slightly and include key quantitative findings (e.g., range of P uptake and soil pH). The abstract mentions significant variations (p<0.05) but does not specified the statistical test used in methodology section.

Lines 38–89: Introduction repeats known concepts. Condense and focus on research gap and hypothesis development.

Lines 47–56: Revise paragraph on phosphorus chemistry to include one or two recent references (2023–2024).

Lines 72–79: Briefly link the P–Zn interaction discussion with the study objectives.

Lines 147–152: Specify version and reference for statistical software (GRAPES).

Lines 90–152: Provide detailed description of soil sampling criteria, replication, and fertilizer treatments. Clarify 150% vs 100% P confusion.

Lines 175–196: Define pfH₂PO₄ and provide units in Table 2.

Lines 226–238: Provide a one-line mechanistic explanation of P–Zn antagonism for clarity.

Lines 226–238: Include two-way ANOVA results for P×Zn interaction.

Lines 242–250: Provide correlation coefficients (r, p) for Figure 3 instead of descriptive statements.

Lines 322–341: Conclusion: Add statement on implications for future field research and sustainability.

**General comments**

Provide correlation coefficient (r) values in Figure 3.

Ensure consistency in statistical notations across all tables

Add references to standard protocols viz. texture by hydrometer method etc..
