# Supplementary material for: Understanding phosphorus dynamics and uptake in diverse soils of Indo-Gangetic Plains of India
Source: PLoS One. 2025 Dec 30;20(12):e0339034. doi: 10.1371/journal.pone.0339034 (PMC12752939; doi:10.1371/journal.pone.0339034)
Supplement: Attachment — Submitted filename: Response to Reviewers.docx [file pone.0339034.s007.docx]

To

The Editor

PLOS One

Ref: Manuscript ID: PONE-D-25-53866

Sub: Submission of revision of the manuscript entitled “Understanding phosphorus dynamics and uptake in diverse soils of Indo-Gangetic Plains of India”

Sir,

Thank you very much for giving me an opportunity to revise the manuscript as referred above.

As per your suggestion, I am herewith sending revised manuscript entitled “Understanding phosphorus dynamics and uptake in diverse soils of Indo-Gangetic Plains of India (Manuscript ID: PONE-D-25-53866)” for your kind consideration for publication in your esteemed Journal “PLOS One”. All the suggestions/corrections of editor and reviewers are incorporated in the revised manuscript. Details of changes we have made are given in the following revision note:

| **Reviewer #1** | |
| --- | --- |
| This manuscript addresses an important topic concerning phosphorus (P) dynamics and uptake in the Indo-Gangetic Plains (IGP), a region of high agronomic significance. The study provides valuable insights into the relationship between soil physico-chemical properties, P availability, and nutrient interactions, particularly the antagonism between P and Zn in wheat. The topic aligns well with the scope of *PLOS ONE*, emphasizing soil fertility, nutrient efficiency, and sustainable crop production.  However, while the study is scientifically sound and the data is extensive, the MS need some minor revisions: | |
| **Title and Abstract** |  |
| The title is appropriate; however, please correct the spelling of *“Phopshorus”* to *“Phosphorus”* in the short title. | Corrected as suggested. |
| In the abstract, the sentence *“excessive P application altered Zn concentration”* may be rephrased as *“**P fertilization altered Zn concentration”* to avoid implying over-application. | Rephrased the sentence as suggested |
| **Introduction** |  |
| The introduction is well written and contextually relevant. Ensure consistent use of “phosphorus (P)” after its first occurrence. | Corrected in the introduction as suggested. |
| **Results** |  |
| In the Tables, please ensure consistent significant figure formatting for numeric data (e.g., 2 decimal places or as appropriate). | Numeric data presented as suggested by the reviewer. |
| **Discussion** |  |
| The discussion effectively relates soil variability and P dynamics and well written while focusing on the most relevant findings. | Thank you for the positive remarks |
| **Conclusion** |  |
| The conclusion is comprehensive. You may consider a concise final sentence emphasizing the applicability of findings to site-specific nutrient management and nutritional quality improvement. | A concise statement was added as suggested. |
| **Formatting and References** |  |
| Check consistency in reference formatting (especially spacing and punctuation between author initials). | Checked and all the references are in Vancouver style as required by the journal. |
| Ensure that all abbreviations (e.g., OC, DTPA) are defined at first use. | All abbreviations are defined in the manuscript. |
| Correct minor typographical errors (e.g., inconsistent capitalization, spacing before parentheses). | Typographical errors corrected wherever found. |
| **Some specific suggestions:** |  |
| Line 92: Check grammar of this sentence. | Sentence corrected. |
| Line 95: Spelling mistake. Need Correction. | Corrected as suggested |
| Line 101: Need Capitalization | Capitalized as suggeted |
| Line 109: this symbol (p) is not in above equation. | Corrected as “µ” |
| Line 125: Combine it as diammonium. | Corrected as suggested |
| Line 113, 114, 120: Write properly by using subscripts. | Corrected as suggetsed |
| Line 127: Treatment combinations are not defined clearly, neither in a table or in-text. | Treatment details added in the sub heading “Greenhouse pot experiment” |
| Table 1: Maintain the uniformity of digits after decimal through the table. Write expanded form of ‘Avail.’ in the footnote of the table. | Decimals maintained uniformly and Avail. Was re written as available. |
| Table 2: Write expanded form of ‘CF’ and other variables in footnote of the table | All abbreviations were expanded. |
| Table 3 &4: Add expanded form and details regarding no. of observations, level of significance, etc. about CV, LSD, and SE(m) terms in table footnote. | Expanded forms added. |
| **Overall Assessment** |  |
| The manuscript is well-structured, data-rich, and scientifically sound. Only minor editorial and formatting improvements are needed. The findings have clear implications for nutrient management in the Indo-Gangetic Plains and merit publication after these small revisions. | |

| **Reviewer #2:** | |
| --- | --- |
| The manuscript 'Understanding phosphorus dynamics and uptake in diverse soils of Indo-Gangetic Plains of India' presents novel insights into phosphorus dynamics and nutrient interactions in wheat under diverse soil conditions. The work is generally sound and within the scope of PLOS ONE. The manuscript is well written and structured logically. The objectives are clear and align with the presented results. However, a few editorial and methodological clarifications are required. Figures and tables could benefit from consistent formatting, and certain results need quantitative support. Key issues include unclear fertilizer protocols, limited explanation of sampling design and interpretation is largely descriptive. | |
| **Line-by-Line Comments** | |
| Lines3: Correct "Phopshorus" to "Phosphorus" in the short title. | Done |
| Lines 36-37: Add "wheat" and "zinc deficiency" for better discoverability. | Added as suggested |
| Lines 20–35: Abstract: Condense slightly and include key quantitative findings (e.g., range of P uptake and soil pH). The abstract mentions significant variations (p<0.05) but does not specified the statistical test used in methodology section. | Added. And methodology mentioned in statistical analysis section. |
| Lines 38–89: Introduction repeats known concepts. Condense and focus on research gap and hypothesis development. | Introduction modified as suggested. |
| Lines 47–56: Revise paragraph on phosphorus chemistry to include one or two recent references (2023–2024). | Recent references added and paragraph was revised. |
| Lines 72–79: Briefly link the P–Zn interaction discussion with the study objectives. | Added extra sentences as suggested. |
| Lines 147–152: Specify version and reference for statistical software (GRAPES). | Specified and reference was added. |
| Lines 90–152: Provide detailed description of soil sampling criteria, replication, and fertilizer treatments. Clarify 150% vs 100% P confusion. | Details are added under subheading “Greenhouse pot experiment” as desired |
| Lines 175–196: Define pfH₂PO₄ and provide units in Table 2. | It is a log value and has no units. |
| Lines 226–238: Provide a one-line mechanistic explanation of P–Zn antagonism for clarity. | Added as suggested |
| Lines 226–238: Include two-way ANOVA results for P×Zn interaction. | Because zinc levels varied naturally among soils and were not applied as experimental treatments, Zn could not be treated as an independent factor. Therefore, a one-way ANOVA was used, as only phosphorus treatments (control vs. 100% P) were imposed experimentally. |
| Lines 242–250: Provide correlation coefficients (r, p) for Figure 3 instead of descriptive statements. | Added as suggested. |
| Lines 322–341: Conclusion: Add statement on implications for future field research and sustainability. | Added as suggested. |
| General comments |  |
| Provide correlation coefficient (r) values in Figure 3. | Given as suggested |
| Ensure consistency in statistical notations across all tables | Corrected as suggested. |
| Add references to standard protocols viz. texture by hydrometer method etc.. | References for standard protocols added. |

| Reviewer #3: |  |
| --- | --- |
| The authors aimed to explain the dynamics of phosphorus and its uptake in various soil types across the Indo-Gangetic Plains of India. The availability of phosphorus fertilizers remains a major challenge for farmers in India. This study contributes to a better understanding of phosphorus fixation and its availability to plants. | We thank the reviewer for their positive and encouraging comments. We appreciate the acknowledgment of the scientific relevance and contribution of our study. As noted, phosphorus availability and fixation remain major challenges for farmers across the Indo-Gangetic Plains, and our work was specifically designed to address these constraints by examining soil variability, P dynamics, and crop uptake patterns. |
